# Co-developing SHELTER (Safe, Healthy Environments and Local Transformation for Equity and Resilience) with families with lived experience of homelessness in the New York City shelter system: A community needs assessment and data collection protocol

**DOI:** 10.1371/journal.pone.0341718

**Published:** 2026-01-28

**Authors:** Diana Margot Rosenthal, Kate Guastaferro, Jasia Kubik, Melody Goodman

**Affiliations:** 1 Department of Social and Behavioral Sciences, School of Global Public Health, New York University, New York, New York, United States of America; 2 Robert F. Wagner School of Public Service, New York University, New York, New York, United States of America; 3 Department of Biostatistics, School of Global Public Health, New York University, New York, New York, United States of America; University of Missouri School of Medicine, UNITED STATES OF AMERICA

## Abstract

In January 2025, the nightly census revealed that over 120,000 people were staying in New York City (NYC) shelters, including more than 41,000 children, of whom almost half were aged 0–5 years. Children under five years old (under-5s) experiencing homelessness are especially vulnerable because the first five years of life are a critical period for child growth, including approximately 90% of brain development. Furthermore, under-5s experiencing homelessness have a higher risk for multiple adverse childhood experiences, developing chronic health conditions, and recurrent homelessness across the life course. Data available for under-5s experiencing homelessness is generally lacking, and what is available is of notably poor quality in the United States, leaving a wide evidence gap and an inability to determine the actual needs of this population. This proposed protocol employs community-based participatory research and was co-developed with families with under-5s who have lived experience of homelessness in NYC shelters. The aim is to determine what barriers exist in the physical and social environments to optimizing health and wellbeing (e.g., milestones, child mental health, parental mental health, safety) among under-5s living in NYC shelters. Using a sequential mixed-methods design, we propose to address a gap in the current literature by conducting an assets- and deficits-based health needs assessment comprising a quantitative survey and qualitative semi-structured interviews. In the long term, our objective is to enhance the quality and quantity of data for this vulnerable population, thereby laying the groundwork for the future co-development of a comprehensive, optimized intervention addressing the needs of under-5s experiencing homelessness.

## 1. Introduction

### 1.1. Background and rationale

Child homelessness is an epidemic impacting over 440,000 infants and toddlers (ages 0–3 years) each year in the United States (U.S.) [1]. In New York City (NYC), the shelter population is comprised of approximately 120,000 individuals, with over 90% identifying as part of minoritized ethnic groups, and approximately one-third are children (ages 0–17 years) [2–4]. The total number of children in families experiencing homelessness and sheltered in NYC by the Department of Homeless Services (DHS) skyrocketed by approximately 120% between 2022 and 2025 [5,6]. This exponential growth may be attributed to the rise of unaffordable housing, an increased number of evictions, and the asylum seeker state of emergency [7,8]. Child homelessness in the early years is an escalating public health concern. Children under the age of five years experiencing homelessness are especially vulnerable because the first five years of life are a critical period for child growth, including approximately 90% of brain development [9–11]. Furthermore, these children have a higher risk for multiple adverse childhood experiences (ACEs), developing chronic health conditions, and recurrent homelessness across the life course [11–13].

Although there is a documented large proportion of children ages 0–4 years (referred to herein as under-5s) experiencing homelessness, the true prevalence remains unknown, as does its long-term impact on child health and development. Data collection on this under-5s population is generally lacking, and what is available is of notably poor quality in the U.S., leaving a wide evidence gap and an inability to determine the actual needs of this population [14,15]. Population estimates are also likely to be underestimated due to differential definitions of homelessness for service eligibility criteria [11,16–18]; the fact that the DHS is not the only shelter system for NYC families [14]; inconsistent reporting periods (e.g., Point-In-Time vs. Cumulative Totals) [19]; inconsistent reporting of age groups among systems-level organizations (i.e., ages 0–3, 0–4, 0–5, vs. 0–17 years) [1,20,21]; and underrepresentation of under-5s in early childhood services data reporting [15] because many services report exclusively on the 0–3-yr age group [1], thereby missing an entire year of critical development.

The Office of the NYC Comptroller recently reported various data limitations, including the fact that the DHS does not track its clients who receive mental health or medical services. These limitations may be attributable to the absence of an integrated data system across different sectors [14] and could highlight potential accessibility issues and service gaps. Additionally, there is limited or non-transparent data collection on asylum seekers [14], which may render them invisible to health services, creating another evidence gap. Without comprehensive and reliable data, we cannot accurately determine the actual needs of this population. Therefore, we cannot inform decision-making or develop targeted interventions to address those specific needs. In October 2023, the National Alliance to End Homelessness emphasized the need for data reform to improve data quality regarding resource gaps and service delivery improvements. One resulting suggestion was to utilize more mixed-methods data to inform practices and guidelines [22], a gap that the current study is trying to address.

Though the current published literature on the under-5 age group is scarce [11,16], significant evidence demonstrates that homelessness and mental health are intrinsically linked [23,24]. Evidence for this association among the under-5 age group, however, is mainly found in non-empirical media. For example, in a Health x Housing Lab webinar, families with lived experience discussed key critical health issues and service gaps they had experienced with their children while living in the NYC shelter system [25]. Families indicated their health needs were neither assessed during shelter intake, nor were they informed about the health services available to them. Health providers reportedly often lacked training to work with children and families experiencing homelessness and did not have knowledge of housing resources [25]. One webinar panelist with lived experience of homelessness specifically mentioned her mental health challenges while living in the shelter system and the impact on her child under age 5: she publicly stated that children feel what their parents feel, and although under-5s do not have the same responsibilities (e.g., paying bills), they have their own problems [25]. This panelist referenced the current gap in mental health services for under-5s, which two other parents corroborated [25]. According to the Mayor’s Office of Community Mental Health, there is increasing demand for mental health screenings and services in NYC, with a referral rate as high as ~80% of families screened for “behavioral health (mental health and substance use) needs,” but as low as 0.4% actually attend an appointment when referred [26,27]. In 2023, a new law mandating mental health services in DHS shelters [28] showed promise in improving care accessibility for families, although no funding or infrastructure was designated to support its implementation [29]. The practical impact of this law has yet to be fully realized; however, concerns have arisen regarding staffing, privacy issues, inadequate Wi-Fi for telehealth, and a lack of predetermined budget allocation [29–31]. Therefore, many preexisting and new mental health issues likely remain unaddressed, which may impact the child’s health and wellbeing while living in a shelter.

Families in NYC also discussed environmental hazards within shelters and the effects on children’s health, including their children not meeting developmental milestones, or getting a nutritious diet with only frozen, processed food provided in the shelter system [25]. The average length of stay in shelters is more than a year, and data are not collected for shelter reentry unless it occurs within a year of exiting. The first five years are vital to development. If some children spend their crucial early years in suboptimal environments and some are even born into them, there is likely to be an impact on their health and wellbeing. Furthermore, the lack of follow-up of children after they leave the shelter system (or return after ≥ 1 year later) is also a critical gap and a missing opportunity to follow those children who have slipped through the net. Few studies have focused exclusively on this marginalized group in high-income countries, nor have they examined these issues using mixed methods, including peer-led inclusive research and collaboration with individuals who have lived experiences. Given these health inequities and the prolonged duration families spend residing in shelters, more research is needed on this less visible population, which requires community-engaged approaches. This project proposes to answer this call, building on our team’s prior research [11].

### 1.2. Current study

The initial aim of this ongoing mixed-methods and community-based participatory research (CBPR) study was to determine what barriers exist to accessing specific health services (e.g., mental health) and optimizing health and wellbeing for families with under-5s who are experiencing homelessness/have experienced homelessness in the NYC shelter system. Research that is not grounded in relevant theories and alienates the lived experience of “*…affected communities in the conception, design, implementation, and dissemination stages will cause more harm than good*” (Sealy-Jefferson 2022, p. 3) [32], which calls for research questions that are not bound by only available data and academic curiosity [32].

This project is co-developed with families who have lived experience in the NYC shelter system (i.e., lived experience experts), acting as citizen scientists and learning transferable skills (e.g., project design, critical thinking, data interpretation, collaboration, intervention development, and publication) while being compensated for their time. The overarching objective is to enhance the quality and quantity of data currently lacking for this vulnerable and transient population, which may inform recommendations to strengthen data collection and ultimately improve their quality of care. This project presents a vital opportunity to track those children who have exited shelters and may be invisible to services. The product of this project will ultimately be a co-developed community-engaged intervention of their choice to address the barriers identified during the needs assessment.

### 1.3. Formative and refinement stage

The project commenced in September 2024, when we established a community advisory board comprising two distinct committees. We formed a lived experience experts (LEEs) advisory committee consisting of parents/caregivers and another advisory committee of key professionals and decision-makers, each with nine members. All advisory committee meetings were hosted virtually on Zoom to ensure accessibility and convenience for our committee members.

The LEEs advisory committee first met with the Principal Investigator (PI) in October 2024, during which the committee was presented with potential research questions (S1 Appendix) informed by prior discussions with families, including the webinar [25]. The LEEs committee met three times online from October to December 2024, with continuous collaborative dialogue via email and a private Slack channel. During the LEEs committee meetings, the research questions (S2 Appendix), specific objectives, and participant eligibility criteria were discussed and refined, thereby co-developing the protocol. Based on prioritization, the main aim was collectively refined to determine: “What barriers exist in the physical and social environments to optimizing health and wellbeing (e.g., milestones, child mental health, parental mental health, safety) among under-5s living in NYC shelters?” Based on the research questions and sub-questions (S2 Appendix), the corresponding mixed-methods study design was developed. During these meetings, we also discussed the appropriate data collection tools (i.e., surveys and semi-structured interviews). We reviewed validated measures, selecting the ones for use in this exploratory study to ensure the feasibility and acceptability of the research among our target population.

In November 2024, our team met with an advisory committee of key professionals and decision-makers in relevant cross-sector fields (e.g., academia, housing, healthcare, social work) to gather feedback on the study design and data collection tools. Both committees met jointly in December 2024 and approved the final quantitative survey, including corresponding research questions and hypotheses, as well as preliminary qualitative questions. We finalized the protocol and co-developed our project title: *“Co-developing SHELTER (Safe, Healthy Environments and Local Transformation for Equity and Resilience)”.* The following protocol outlines the ongoing and future activities of this exploratory study, designed to collect initial data to determine which research questions warrant further investigation. We plan to use the data and sustain work with community partners to refine the research questions for a future larger study.

## 2. Methods

### 2.1. Study design

The project takes place in NYC, New York, U.S. This exploratory study follows an Equal-Status Sequential mixed-methods design, giving equal weight to our quantitative survey and qualitative semi-structured interview data [33]. Mixed-methods designs integrate quantitative and qualitative components, comprising viewpoints, data collection, analysis, and/or interpretation, alongside a framework that facilitates the amalgamation of these components [34,35]. Mixed methods were selected for this project because they complement pragmatism by enabling researchers to engage with the community, focus on “what works,” and study the same phenomena through multiple experiences [36,37]. This approach directs the inquiry toward adaptation and problem-solving, validating research questions, and ensuring practically relevant findings [36,37]. CBPR was selected because this collaborative approach to research aims to ensure that the research process is inclusive, equitable, and grounded in the real-world experiences of the community. CBPR is a partnership between researchers and community members to address complex issues that directly affect the community [11,32,38,39].

This research and its aim are theoretically grounded in and guided by the Nurturing Care Framework (NCF) [10] and the Social Ecological Model (SEM) [40]. The NCF includes five components, which are required for children to optimize their full potential: 1) good health; 2) adequate nutrition; 3) security and safety; 4) opportunities for early learning; and 5) responsive caregiving, plus the enabling environments for nurturing care [10]. At each level (i.e., child, family/caregiver, community, culture & society, policy, and systems), the SEM [40] helps illustrate protective and risk factors that either build or hinder a conducive environment that enables families and caregivers to provide nurturing care to their children [10]. Enabling environments at each level include the caregivers’ capabilities, empowered communities, supportive services, and enabling policies [10]. The SEM offers a multi-level structure, which is useful for visualizing barriers that may exist in both physical and social environments. Specifically, the NCF and SEM shaped the survey and interview design as well as the *a priori* codes for the qualitative analysis.

### 2.2 Ethical considerations

The full protocol has been approved by the Institutional Review Board (IRB) at New York University (IRB-FY2025–9678) and registered on the Open Science Framework (OSF): osf.io/yb6u9 [41]. All university-based research personnel have ensured their training and certification with the Collaborative Institutional Training Initiative (CITI) Program is up to date as required for research with human subjects in the U.S. They have also completed Child Safeguarding Training provided by UNICEF [42]. The PI and lead Research Assistant, a graduate student, were trained in “Providing Trauma-Informed Care in Homeless Response” by the National Alliance to End Homelessness [43]. They developed action plans to ensure adherence to the six key principles of trauma-informed care during the project.

Given the vulnerability of participating families, appropriate safeguards must be in place to protect their rights and welfare. The PI consulted with the advisory committees and project collaborators and, together, compiled an extensive list of external resources available to participants during the project, such as counseling, childcare, and housing advice. Consent forms, protocols, information sheets, and recruitment materials were reviewed by the IRB and the advisory committees to ensure they are comprehensible to potential participants. As advised, many families may not want to sign their names on the consent form due to confidentiality concerns. Therefore, we secured a waiver of signed consent. All potential participants will be given a consent form with a project information sheet, which includes the PI’s contact information, the list of external resources, and details on how the participant's identity will be strictly protected. These forms will state that participation in the project is entirely voluntary, and non-participation will not affect the client’s benefits or services. Furthermore, participating clients can withdraw at any time without affecting their benefits or services.

### 2.3. Participants

#### 2.3.1. Recruitment.

Due to the transient nature of families living in the shelter system, obtaining a fully representative sample will be challenging. We will mitigate this by utilizing various recruitment routes, including a settings-based approach, such as family health clinics, child welfare programs, shelters, and charities that are regularly accessed by this population. We will work with both advisory committees and utilize snowballing to identify potential participants. We have also partnered with NYC-based organizations and shelters to support our recruitment efforts, with materials distributed by staff at their respective sites. Using public Global Positioning Systems (GPS) data and with the help of the advisory committees and partners, we will map salient neighborhoods across NYC that have family shelters or provide aid to families experiencing homelessness to target our study advertising efforts. Research assistants (RAs) will travel to these locations (e.g., Sunset Park) to post flyers and distribute postcards in public areas, particularly outside shelters and schools. All recruitment materials include a QR code linked to the survey and our research team’s email address, which potential participants can use to ask questions or request assistance with completing the surveys. We will also advertise the study on a NYC podcast run by families with lived experience of homelessness, which is widely recognized within their communities.

#### 2.3.2. Inclusion and exclusion criteria.

Eligibility will require participants to be at least 18 years old and have at least one child under 5 years old currently living with them in a shelter in NYC, or have had at least one child under 5 years old living with them in a shelter in NYC at any time from 2008 to the present. The 2008 cutoff was selected because any earlier timeframe would mean their child would be an adult (≥ 18 years old) in 2025. To mitigate recall bias, we will use standardized questions with validated metrics, which we have already piloted with this demographic. We will also review shelter experiences by date to see whether these have changed over time, reflecting any policy changes.

Participants will need to confirm that their shelter is or was located in NYC by supplying the zip code or, to promote inclusivity, the zip code of the nearest landmark. Eligibility screening in the survey instrument will ask participants to self-confirm that they meet the criteria. The inclusion criteria will also include the McKinney-Vento Homeless Assistance Act definition of homelessness for the context and setting of this study [44]. However, due to the proposed purposive and convenience sampling methods, the study results will not be generalizable to all families with children of all ages experiencing homelessness who also fall under this federal definition.

#### 2.3.3. Sample size.

For the quantitative component, convenience (purposive) sampling will be used to represent our population of interest (under-5s living in shelters and those who have exited) [33]. The sample size was estimated using G*power software (α = 0.05; Df = 4; Power = 0.8) [45,46]. Based on the chi-squared (χ2) test and our variables of interest, we determined that a range effect size |p| = 0.3–0.4 results in our target sample size ranging from 75 to 133 participants [47]. Recruitment will not exceed 400 individuals, including eligible and non-eligible participants [48].

The qualitative component will involve a purposive sample of families living in shelters and those who have exited, ensuring we recruit participants who can answer the interview questions from both perspectives. We will have previously noted their housing status during the survey compensation process. Approximately 20–30 survey participants will be recruited, or until data saturation is reached, meaning that as we collect data, no new themes emerge in the analyses [49]. This sampling strategy is not only pragmatic for our population of interest, given their high mobility, but also enables us to deliver rich, in-depth data about this specific population.

### 2.4. Study procedures

Study recruitment was launched on May 7, 2025. The targeted date for finalizing recruitment is September 30, 2025; however, qualitative data collection will continue through November 2025. The study is a community health needs assessment using deficit- and asset-based approaches, thereby identifying the community’s needs and gaps, as well as its strengths and capacities [50]. “Community” will be defined by ensuring diverse representation and recruiting participants from various Department of Homeless Services (DHS) and other certified family shelters of all sizes across all five boroughs, identified either directly from provider lists [51] or through referral. The LEEs advisory committee has connections within several shelters, including domestic violence (DV) shelters. The total number of shelters in New York City is not publicly available and fluctuates due to ongoing policy changes. Additionally, accurate shelter counts are complicated by the inclusion of safe havens and DV shelters.

Surveys will be administered in English and Spanish on the secure online platform REDCap [52,53]. We discussed the advantages and disadvantages of conducting the survey online with our community advisory board, and the consensus was that most families should have internet access (post-pandemic) and at least one Wi-Fi-enabled device (e.g., mobile phone, tablet, laptop). Overall, this data collection method was considered the most convenient for the parties involved. Almost half of the families experiencing homelessness in NYC are Hispanic or Latino [3], so we are offering the survey in Spanish to accommodate this demographic and prevent excluding almost 50% of the population from the study. Due to resource constraints, we will only offer the translated survey materials in these two languages. Participants will complete the survey independently, but accommodations will be made for potential participants with lower literacy or English as a second language. Our bilingual team will also be available to assist them in filling out the survey upon request, either in person, over the phone, or online via Zoom. We will also offer these options to mitigate digital exclusion due to poor Wi-Fi in shelters or a lack of consistent access to a smartphone or computer.

Potential participants will be provided the consent form in REDCap and must provide informed consent by selecting “Yes” on the form. Eligible participants will be automatically directed to one of two survey versions depending on their shelter status, as described below (**2.5 Measures**). The surveys will take approximately 25–30 minutes to complete and can be saved for completion at a later time by providing an email address. All data will be de-identified during the survey verification and participant compensation stages.

As the literature recommends, preventive measures available in REDCap (e.g., picture and word CAPTCHA) will be used to prevent bot entries [54]. Additional screening for bots (i.e., non-human respondents) will be conducted using random “challenge questions” placed sporadically in the surveys (e.g., “Which of the following is a vegetable?”, “Which of the following numbers is highlighted?”) as well as fill-in-the-blank text questions [54]. The research team will verify all survey entries to ensure the quality of the data. Common patterns of bot or falsified entries (e.g., multiple sequential registrations within seconds of each other, participant's age is not feasible with the reported child’s age [e.g., 1-yr age difference between biological parent and their child], zip codes outside of the eligible area) will be manually flagged by the research team for a thorough review. We will also manually double-check the participant’s inclusion criteria and survey completion time. If we determine that they are not following study procedures or that they may be providing false information deliberately, we may terminate their participation in the study early. For incomplete surveys, we may contact respondents (if an email address is provided) to offer an opportunity to complete the survey. If we are unable to reach them and/or they do not complete the survey, they will be excluded.

At the end of the quantitative survey, participants will be asked whether they would like to participate in an optional interview, during which we can ask more in-depth questions about their experiences in the shelter system. If they would like to participate, we will contact them via email to arrange a time for the semi-structured interview. Semi-structured interviews are focused yet flexible and can be guided by participants’ responses, allowing for greater in-depth exploration than quantitative data in an iterative way [55]. We will use the preliminary survey data to create a qualitative interview topic guide, which the community advisory board will review. The topic guide will be piloted on a few participants first to ensure the open-ended questions are clear (some preliminary questions are in the S2 Appendix).

For the qualitative component, the PI will conduct all semi-structured interviews in English or Spanish (with a bilingual student RA as an interpreter), either in person, by phone, or via Zoom at a time convenient for the participant. Interviews will last approximately 30–45 minutes and will be terminated if the 1-hour limit is reached. The interview will be recorded and then de-identified. Interviews will be recorded online on Zoom, and a handheld voice recorder will be used for in-person or phone interviews. After we check the transcripts for accuracy against the audio recordings, we will delete the recordings from Zoom and the handheld recorder within three business days. Furthermore, the interviews will not be linked to individual participant survey data.

### 2.5. Measures

We will ask participants to complete a quantitative survey regarding their experiences living with their children, who are/were under the age of 5, in the NYC shelter system. There will be two versions of the survey, administered based on current living situation: one for those currently living in shelters and one for those who have already exited. These surveys will utilize the same validated measures and questions, with the language slightly adjusted to reflect their specific circumstances (i.e., shelter status). In consultation with both advisory committees, we determined that this approach would be the most effective way to alleviate participant burden, making it easier for participants to understand the questions. We have consulted with external community-based organizations (e.g., advocacy groups, family health centers, shelters, charities) and NYC Agencies & Departments serving the homeless population in NYC regarding our research methods. These consultations ensure we will meet our overarching objective of improving the quality and quantity of data.

Before recruitment, surveys were piloted on REDCap [52] with our LEEs advisory committee members. After the pilot, we met with four advisory committee members to gather feedback on their experiences with the survey. Following this, we made further refinements to improve the online survey’s accessibility and usability, ensuring the questions were clear and relevant to the target population. The surveys will include six sections related to our theoretical orientation (NCF and SEM) and research questions: socio-demographics, housing history, physical environment, social environment, child health, and parental mental health. **Table 1** links each section to the corresponding measures, research questions, and NCF components; measures with an asterisk (*) indicate questions with slight adaptations, including more inclusive answer choices to reflect our population. (Please consult S3 Appendix for the survey that will be administered to families who have already exited shelter, which pertains to the tools/instruments and questions ^**±**^ outlined in **Table 1**.)

**Table 1:** Mapping of survey measures to the theoretical framework and research questions.

Domain	Measure	Description	Tool/Instrument (Survey Question #) ^±^	Corresponding Research Question(s)	NCF Component
**Socio-Demographics**	Child characteristics Parent/carer characteristics Parent/carer relationship to child Language Insurance coverage Household size	Basic information such as age, gender, race*, employment*, and socioeconomic status* Place of birth and primary language spoken in household* English proficiency Insurance provider (or lack thereof) Enrollment in government assistance programs Number of children per age group living in household*	National Survey of Child Health (NSCH) **(Q1-22)** U.S. and U.K. Censuses **(Q3, 6-10, 14, 19, 21-22)** Homeless Health Needs Audit **(Q19a)**	Are certain demographics more prevalent among families with under-5s living in shelters, and compared to the general population?	Good health Responsive caregiving Security and safety
**Housing History**	Duration in shelter Number of shelter stays Current sleeping situation Reasons for homelessness	Length and frequency of shelter stays and moving* Type of shelter or housing accommodation* (if exited) they currently reside in Primary, secondary, and optional additional reasons for the most recent time they became homeless* (e.g., financial problems, domestic violence, mental or physical health problems)	Homeless Health Needs Audit **(Q23-28)** ACORN screening tool sourced from the Social Interventions Research and Evaluation Network (SIREN) **(Q27)**	Is there an association between the physical and social environments of shelters and child growth and development?	Security and safety
**Physical Environment**	Space to play Shared spaces Presence of mold/vermin Neighborhood safety Access to outdoor spaces Internet access	Amount of space for child to play (inside/outside)* Details about shared spaces in communal living* Presence of internal and external health risks (e.g., unsafe electrics in accommodation, secondhand smoke, mold, pests/vermin)*	“Statutory homelessness in England: the experience of families and 16-17 year olds" survey **(Q29-52)** Kingfisher housing evaluation **(Q31-34, 38, 41-45, 47, 49)** National Survey of Child Health (NSCH) **(Q29, 36, 38, 41-42, 47, 50, 52)**	Is there an association between the physical and social environments of shelters and child growth and development?	Good health Security and safety Responsive caregiving
**Social Environment**	Number of social interactions Number of outside activities Number of books/toys Hours of screen time	Opportunities for children to socialize with family/community members Access to early learning and developmental growth in shelter	Home Observation for Measurement of the Environment (HOME) **(Q53-62)**	Is there a relationship between the physical and social environments of shelters and poor mental health (both parent and child)?	Responsive caregiving Opportunities for early learning
**Child Health**	Family health history Child’s health as an infant Adverse Childhood Experiences (ACEs) Food security Developmental milestones Healthcare access	Family health history (e.g., chronic health conditions*) Child’s health information before, during, and after (if applicable) in shelter* to ascertain whether they are meeting developmental milestones or have been negatively impacted by shelter environment Level of food security to ascertain whether child is receiving adequate nutrition To what extent healthcare needs are satisfied within shelter	National Survey of Child Health (NSCH) **(Q64-71, 73-87)** 2019 NYC KIDS Survey **(Q64-65, 67, 81-86)** U.S. Household Food Security Survey Module **(Q72)**	Are certain developmental delays or childhood ailments more prevalent in under-5s living in shelters? Is there an association between the physical and social environments of shelters and child growth and development? Is there a relationship between parental mental health and child growth and development? Is there a relationship between the physical and social environments of shelters and poor mental health (both parent and child)?	Good health Adequate nutrition
**Parental Mental Health**	Depression Anxiety Adverse Childhood Experiences (ACEs)	Additional variables of interest (e.g., quality of life, psychological wellbeing)	Patient Health Questionnaire (PHQ-8) **(Q88)** General Anxiety Disorder (GAD-7) **(Q89)** World Health Organization (WHO) **(Q90)**	Is there a relationship between parental mental health and child growth and development? Is there a relationship between the physical and social environments of shelters and poor mental health (both parent and child)?	Good health

* Adapted measure.

± (Survey Question #) corresponds to the question numbers in the survey for families who have exited shelter (S3 Appendix). Please note that some question numbers overlap, as wording or answer choices were merged from different tools.

Participants will be asked to provide socio-demographic information on their household (e.g., primary language, household income), themselves (e.g., education, marital status, employment status), and their under-5s (e.g., ethnicity, age, gender, benefits coverage), primarily using the National Surveys of Child Health (NSCH) [56–59]. Housing history will be ascertained using the Homeless Health Needs Audit [60]. Questions include primary and secondary reasons for homelessness from the participant’s perspective, duration of living in a shelter, number of shelter stays, and current sleeping situation. We adapted the reasons for homelessness categories during the initial meetings with the advisory committees and provided detailed explanations under each prompt. The current sleeping situation question was adapted by providing different categories of shelters in NYC (e.g., emergency accommodation, hostel, DHS shelter, domestic violence shelter, Department of Housing Preservation and Development shelter, Health and Hospitals (H + H) shelter, etc.).

The survey version for those who have exited the shelter system includes more categories for the current sleeping situation to better reflect those who are no longer living in NYC shelters. For example, an owner-occupied home, a rental apartment, house, or room (with or without government subsidy), sofa surfing, or staying with friends or relatives. We adapted the categories from the Homeless Health Needs Audit [60] and the ACORN screening tool sourced from the Social Interventions Research and Evaluation Network (SIREN) [61,62]. For participants who have exited the shelter system, we will also request the approximate date of their exit.

The conditions of the physical environment will be determined using the [60] Kingfisher housing evaluation [63], NSCH [56], and adapted temporary accommodation prompts from “*Statutory homelessness in England: the experience of families and 16-17 year olds* [64].*”* Questions will pertain to the internal and external shelter environment. Participants will list the number of household members by age who share a room as their bedroom. Using this data, we will calculate an overcrowding metric, either the Bedroom Standard or the Persons per Bedroom ratio [65]. Families will also indicate whether they share kitchen, laundry, and bathroom facilities with people who are not members of their household. On a five-point Likert scale from “Never” to “Always”, participants will answer how often they have trouble in their accommodation with hazards such as dampness (e.g., condensation), mold, vermin (e.g., mice, bedbugs, cockroaches), or unsafe electrics (e.g., electrical units coming out of the wall, lack of baby/childproofing). The surveys will also ask if participants have access to a safe outdoor space for their children to play and exercise, and if not, whether they have access to a nearby park. Several questions will include binary (Yes/No) responses to minimize survey fatigue for the participants. Examples of these questions will include internet access, perceived safety of the neighborhood, overcrowding, problems with passive smoking, and access to a supermarket within easy reach of the shelter accommodation.

Participants will be asked about their social environment and lifestyle using the Home Observation for Measurement of the Environment (HOME) [66]. Most questions ask about the frequency of social interactions, including community and family support, with options such as “Never,” “Once or twice a month,” “More than once a week,” “A few times a year,” “Once a week,” and so forth. Example interactions will include how often their child eats a meal with family members, how frequently their family attends religious services, how often the whole family gets together with relatives or friends, whether the child has a chance to get out of the house, and whether an adult reads stories to the child. Participants will also be asked how many books and toys their children have, and how many hours of TV or other screens are used in their home daily [67]. We examined how Lansford *et al.*(2022) [67] updated the HOME measure and discussed the modifications with the community advisory board. Guided by the review and their feedback, we tailored the questions to align more closely with the needs of our study population.

In the child health section, respondents will report on the child’s health as an infant, including milestones and delays, access to health services, adverse childhood experiences (ACEs), and family health history, using the 2023–2024 NSCH [56,57] and the 2019 NYC KIDS Survey [68]. To be more inclusive and comprehensive, we added more chronic health conditions to the family health history (e.g., tuberculosis, sickle cell disease, and seasonal allergies [different from asthma]), and provided explanations (e.g., hypertension is defined as high blood pressure). For those who have exited the shelter system, we will inquire about the child’s overall health while living in shelter and their current health status. Families will also report on food security using the six-question U.S. Household Food Security Survey Module [69]. For those currently in the shelter system, we will ask about food security over the last 12 months. For those who have exited, we will ask questions about their time living in a shelter more generally. For child mental health, respondents will be asked how true the following statements are regarding their child (e.g., “Your child is affectionate and tender with you”; “Your child bounces back quickly when things do not go his or her way”) [57]. In addition, they will be asked whether their child has received any treatment or counseling from a mental health professional. Lastly, eleven ACEs will be assessed using the NSCH [57,70].

According to the NCF, parental mental health plays a vital role in child development. Parental mental health will be assessed using the Patient Health Questionnaire (PHQ-8) for self-reported depression [71], the Generalized Anxiety Disorder 7-item scale (GAD-7) for self-reported anxiety [72], and the ACEs questionnaire from the World Health Organization (WHO) [73]. We removed any specified time and generalized it to the respondent’s time in shelter, e.g., “During your time in shelter, how often have you been bothered by the following problems?” For the parent’s/caregiver’s ACEs questions, there will be thirteen categories of childhood experiences: physical abuse; emotional abuse; contact sexual abuse; violence against household members; alcohol and/or drug abuser in the household; incarcerated household member; household member treated violently; one or no parents, parental separation or divorce; emotional neglect; physical neglect; bullying community violence; collective violence) [73]. ACEs will be presented as standard binary yes/no responses, with subcategories under each childhood experience, so the respondent does not need to specify which specific adversity they experienced.

For the qualitative component, participants will be asked to answer a series of explanatory questions to help unpack the survey findings. Some questions will be derived from particular phenomena, such as environmental factors, disability, household characteristics, ACEs, or race, identified during the preliminary quantitative findings [33]. In contrast, others (S2 Appendix) have already been co-written with our LEEs advisory committee as previously described (**1.3 Formative and refinement stage**). Participants will provide valuable input regarding their thoughts on the shelter environment and its impact on their child(ren)’s health. Topics will include whether there are developmental delays, such as missed milestones, among under-5s living in shelters, and if so, what types of delays exist. Additionally, the study will investigate whether environmental hazards in shelters impact these delays and, if so, how. Families will be asked about their perceptions of safety in shelters and what prevents a safe and healthy environment, namely, the barriers. From their perspective, we will ask if their basic needs were met while living in shelters and when they exited the system. Lastly, we will ask them about shelter policies and if they have any recommendations for improvement. We will also discuss with participants how the information gathered from them will be analyzed and how the results and feedback will help guide us in co-developing the intervention in the long term.

### 2.6. Incentive structure

Participants will receive a $30 Mastercard gift card for completing the survey. If they choose to participate in an optional interview, they will receive an additional $40 gift card as a token of appreciation for their time and effort in completing the interview. We will require participants to complete at least 75% of the survey questions to receive compensation, thereby allowing them to skip questions that might make them feel uncomfortable. We will end their participation in the study if we are unable to contact them to complete their survey or book their interview (if applicable).

After completing the survey and/or interview, we will require a few business days to process the gift card and verify that the participant followed the study procedures. The gift cards will be sent to them via email through the platform Tango [74] because it will be the fastest way to reach the participant and can be redeemed immediately. Both advisory committees approved this compensation process for its feasibility, and it was used during the pilot without complications. On Tango, we will also verify if participants have redeemed their gift cards and follow up with those who have not to ensure they receive compensation. If participants are unable to access the electronic gift cards due to technical reasons, we will make accommodations to send them a gift card by mail.

## 3. Conclusion

This protocol paper is a comprehensive blueprint for data collection in our mixed-methods exploratory study, Co-developing SHELTER. With increasing numbers of children experiencing homelessness during the current U.S. housing crisis, this issue also remains a growing public health concern; however, there is limited tracking of its impact on children during the most critical years of their development. The quantitative survey and qualitative interview results will help address a broader data gap regarding the health needs of children under age 5 in NYC shelters. These initial data will also guide our next steps with partners, which are likely to include a larger study and contribute to the co-development of interventions to be piloted in NYC. Our study aims to inform future research that prioritizes the co-development of health intervention services for this vulnerable group, using community-engaged approaches with LEEs such as families with lived experience of homelessness. Emphasizing transparency, ethical integrity, and methodological rigor ensures the research yields valuable insights while promoting trust and collaboration among partners.

## Supporting information

S1 Appendix Potential research questions and sub-questions. (PDF)

S2 Appendix Refined research questions and sub-questions. (PDF)

S3 Appendix Survey for families who have exited shelter (post-pilot version). (PDF)
