# Supplementary material for: Co-developing SHELTER (Safe, Healthy Environments and Local Transformation for Equity and Resilience) with families with lived experience of homelessness in the New York City shelter system: A community needs assessment and data collection protocol
Source: PLoS One. 2026 Jan 28;21(1):e0341718. doi: 10.1371/journal.pone.0341718 (PMC12851475; doi:10.1371/journal.pone.0341718)
Supplement: S1 Appendix — (PDF) [file pone.0341718.s001.pdf]

S1 Appendix. Potential research questions and sub-questions

The following potential questions were proposed to the LEEs advisory committee in October 2024, asking which issues were of greater priority to address in this project.

**Potential research questions and sub-questions**
- Are certain socio-demographics or characteristics at higher risk of worse parental mental health among those experiencing homelessness in the shelter system? E.g. asylum seekers, migrants, socio-economic position, ethnicities, disabilities, co-morbidities, LGBTQIA+, household structure (e.g. single parent), victims of domestic violence, insurance coverage, time spent living in the shelter system, pathways to homelessness, pandemic-related, suboptimal living environments.   - How does this affect their child(ren)'s mental health?   - How is this measured (if at all)?   - What services are available for families to access while in the shelter system? Are there services specific to under-5s?   - If there are services for under-5s, are there any barriers preventing families (parents or children [regardless of age]) from accessing these services?   - Are there barriers to accessing services after exiting the shelter system?
- What is the prevalence of children in shelters (past/present) with poor health outcomes?   - Are there delays in developmental milestones? If so, what types of delays?   - Do environmental hazards in shelters impact these, and how? Are parents' choices or behaviors variables? E.g. Not allowing a baby to crawl on the floor because it's dirty or has pests/vermin.   - What is the prevalence of malnourished children?   - This can be broken down into smaller projects, which undergraduate or graduate students might like to get experience with. E.g., anemia, weight, breastfeeding, and vitamin deficiencies   - Food security- pre/post-shelter system- how does this play a role?   - What are common childhood ailments (e.g., fever, respiratory symptoms, accidents, diarrhea) in this population?   *Potentially look at their service uptake, like ER visits, age-specific check-ups and vaccinations.
To what degree is health and wellbeing determined by spatial inequality/inequity? i.e. shelter system placement (random allocation and not convenient to resources, e.g. schools, jobs, pediatricians, services; families' lives uprooted)<br>Could this be determined using citizen science tools and a mapping analysis?
