# Supplementary material for: Co-developing SHELTER (Safe, Healthy Environments and Local Transformation for Equity and Resilience) with families with lived experience of homelessness in the New York City shelter system: A community needs assessment and data collection protocol
Source: PLoS One. 2026 Jan 28;21(1):e0341718. doi: 10.1371/journal.pone.0341718 (PMC12851475; doi:10.1371/journal.pone.0341718)
Supplement: S2 Appendix — (PDF) [file pone.0341718.s002.pdf]

S2 Appendix. Refined research questions and sub-questions

The LEEs advisory committee met with the PI (Dr. Rosenthal) in October 2024 and refined the research questions. Based on these questions, the mixed-methods design was determined: Equal-Status Sequential: (QUAN→ QUAL), giving equal weight to the quantitative and qualitative data results.

**Refined research questions and sub-questions**
<u>**Overarching Question:**</u> What barriers exist in the physical and social environments to optimizing health and wellbeing (e.g. milestones, mental health [including parental mental health], safety) among children under age five (under-5s) living in New York City shelters?
**Quantitative:**
• Is there an association between the physical and social environments of shelters and child growth and development?<br>○ Potential hypothesis: There is a positive association between the physical and social environments of shelters and child growth and development.<br>○ Potential hypothesis: A poorer or suboptimal shelter environment is an independent predictor of poor child health outcomes.
• Are certain demographics more prevalent among families with under-5s living in shelters?<br>○ Potential hypothesis: possible demographics include single women/single-parent households; Black and Hispanic people (Recent influx of migrants within the past few years altered these demographics).
• Are certain developmental delays or childhood ailments more prevalent in under-5s living in shelters?<br>○ Potential hypothesis: possible developmental delays or childhood ailments include<br>▪ Learning to walk (e.g., roaches and vermin on the floor may prevent safe crawling)<br>▪ Colds and respiratory issues<br>▪ Bacterial infections<br>▪ Dormant diseases (i.e., autoimmune diseases) can be triggered by suboptimal conditions within shelter (physical environment and physical and mental stress)
• Is there a relationship between the physical and social environments of shelters and poor mental health (both parent and child)?

- Potential hypothesis: There is a positive association between the physical and social environments of shelters and mental health.
- Potential hypothesis: A poorer or suboptimal shelter environment is an independent predictor of poor mental health.

- Is there a relationship between parental mental health and child growth and development?
  - Potential hypothesis: There is a positive association between parental mental health and child growth and development.
  - Potential hypothesis: Poorer parental mental health is an independent predictor of poor child health and well-being outcomes.

Qualitative:

- What are the families' perceptions of the shelter environment and its impact on their child(ren)'s health?
- Are there delays in developmental milestones among under-5s living in shelters? If so, what types of delays? Do environmental hazards in shelters impact these, and how? Are parents' choices or behaviors variables? E.g., Not allowing a baby to crawl on the floor because it's dirty or has pests/vermin.
- What shelter policies affect how the physical and social environments operate and consequently impact children under 5 and their families?
- What are families' perceptions of *safety* in shelters?
- What prevents a safe and healthy environment in shelters? What are these barriers? (e.g., maintenance issues, food provision, amenities [stove, microwave, hot plates, refrigerator, etc.]).
- From the families' point of view, are basic needs being met while living in shelters and when exiting the system?
- We will ask participants: What are the current policies in shelters (could be local, state, or national level) that impact you and your child?
  - Do you have any policy recommendations based on the issues we've discussed?
  - What safety measures can be taken to mitigate these barriers and prevent suboptimal environments?
- If food insecurity is highly prevalent in the quantitative survey, we will ask more questions from the full 18-question U.S. Household Food Security Survey.

• We will also ask families about the age of the child with respect to the size of their shelter bedroom, e.g., where they upgraded to a larger room when their child turned a certain age.
**Mixed:**
• <u>Study Design:</u> Equal-Status Sequential: QUAN→ QUAL<br>○ Equal weight to the quantitative and qualitative data results<br>• How do the follow-up qualitative findings help explain the initial quantitative results?
