# Supplementary material for: Co-developing SHELTER (Safe, Healthy Environments and Local Transformation for Equity and Resilience) with families with lived experience of homelessness in the New York City shelter system: A community needs assessment and data collection protocol
Source: PLoS One. 2026 Jan 28;21(1):e0341718. doi: 10.1371/journal.pone.0341718 (PMC12851475; doi:10.1371/journal.pone.0341718)
Supplement: S3 Appendix — (PDF) [file pone.0341718.s003.pdf]

S3 Appendix. Survey for families who have exited shelter (post-pilot version)

Socio-Demographics

Researcher's Note -Source: National Survey of Children's Health (NSCH), U.S. and U.K. Censuses, and Homeless Health Needs Audit

We would like to ask you to provide some socio-demographic information about you and your child(ren) who previously lived in shelter. Please select one answer per question.

1. How many children under age 18 years old **currently** are in your home? ▢▢
2. How many of these children are **currently** under five years old? ▢▢

3. What is the primary language spoken in your household?
▢ English
▢ Spanish
▢ Chinese
▢ Russian
▢ French
▢ Haitian
▢ Italian
▢ Bengali
▢ Yiddish
▢ Korean
▢ Polish
▢ Akan
▢ Other language, please specify

In the following questions, "child" only refers to your child(ren) who was under five years old while you lived in the shelter system. Please complete the questions for EACH of your children who were under five years old while you lived in the shelter system.

(Researchers' note: The participant will be able to add additional children to report)

4. What is this child's **current** age?
▢▢ Years (or) ▢▢ Months

5. What is this child's sex?
▢Male
▢Female
▢ Prefer not to say

6. What is this child's race?
▢ Black or African American
▢ American Indian or Alaska Native
▢ Asian Indian
▢ Chinese
▢ Filipino
▢ Korean
▢ Japanese
▢ Vietnamese
▢ Other Asian
▢ Native Hawaiian
▢ Chamorro
▢ Samoan
▢ Other Pacific Islander
▢ Middle Eastern
▢ White
▢ Hispanic or Latino
▢ Prefer not to say
▢ Other—please state:

7. Was this child born in the United States?
▢ No (Please answer questions 7a)
▢ Yes (Please go to question 8)

7a. If not born in the U.S., how long has this child been living in the U.S.?
▢▢ Years and ▢▢ Months

8. Does this child have a disability?
▢ No
▢ Yes

9. Is this child CURRENTLY covered by ANY kind of health insurance or health coverage plan?
▢ No (Skip next question)
▢ Yes

10. Is this child covered by any of the following types of health insurance or health coverage plans?

a. Insurance through a current or former employer or union	▢ No<br>▢ Yes

b. Insurance purchased directly from an insurance company	▢ No<br>▢ Yes
c. Medicaid, Medical Assistance, Child Health Plus or any kind of government assistance plan for those with low incomes or a disability	▢ No<br>▢ Yes
d. TRICARE or other military health care	▢ No<br>▢ Yes
e. Indian Health Service	▢ No<br>▢ Yes
f. Other, specify:	▢ No<br>▢ Yes

Please answer the following questions about your socio-demographics.

11. What is your relationship to the child in the previous questions?
▢ Biological mother/father
▢ Adoptive mother/father
▢ Parent's girlfriend/boyfriend
▢ Foster mother/father
▢ Brother/sister
▢ Aunt/Uncle
▢ Grandmother/grandfather
▢ A different role (child's legal guardian)
▢ Other (not child's legal guardian)

12. What is your age?
▢▢ Years

13. What is your sex?
▢Male
▢Female

14. What is your gender?
▢Man

☐Woman
☐ Non-binary/Gender nonconforming
☐ I identify in another way. Comments (optional) ___________________________

15. Where were you born in the United States?
☐ No
☐ Yes
16. Do you have a disability?
☐ No
☐ Yes

17. What is your marital status?
☐ Married/ civil partnership
☐ Not married, but living with a partner
☐ Not married but in a relationship, living separately
☐ Single
☐ Separated/ Divorced
☐ Widowed
☐Other

18. What is the highest grade or year of school that you have completed?
☐ 8th grade or less
☐ 9th-12th grade; No diploma
☐ High School Graduate or GED completed
☐ Completed a vocational, trade, or business school program
☐ Some College Credit, but no Degree
☐ Associate Degree (AA, AS)
☐ Bachelor’s Degree (BA, BS, AB)
☐ Master’s Degree (MA, MS, MSW, MBA)
☐ Doctorate (PhD, EdD) or Professional Degree (MD, DDS, DVM, JD)

19a.Which of these categories best describes you at present?
☐ Going to school or college full-time (including on vacation)
☐ In paid employment or self-employment (or away temporarily)
☐ Doing unpaid work or voluntary work
☐ Intending to look for work but prevented by temporary sickness or injury
☐ Permanently unable to work because of long-term sickness or disability
☐ Unemployed and not looking for work
☐ Unable to work because of childcare responsibilities
☐ Unemployed and looking for work
☐ Other -- please state:

19b. Were you employed at least 50 out of the past 52 weeks?
▢ No
▢ Yes

20. Was your household income under $20,000 in the last calendar year (January 1-December 31, 2024)?
▢ No
▢ Yes

At any time DURING THE PAST 12 MONTHS, even for one month, did anyone in your family receive...

Cash assistance from a government welfare program?	▢ No<br>▢ Yes
Food Stamps or Supplemental Nutrition Assistance Program (SNAP) benefits?	▢ No<br>▢ Yes
Free or reduced-cost breakfasts or lunches at school?	▢ No<br>▢ Yes
School meal debit/Electronic Benefits Transfer (EBT) cards?	▢ No<br>▢ Yes
Benefits from the Women, Infants, and Children (WIC) Program?	▢ No<br>▢ Yes

21. How good are you at speaking English when you need to in daily life, for example, to have a conversation on the telephone or talk to a professional such as a teacher or a doctor?
▢ Very Good
▢ Good
▢ Fair
▢ Poor
▢ Very Poor

22. How good are you at reading English? For example, immunization pamphlets, health forms for school, and shelter intake forms.
▢ Very Good
▢ Good
▢ Fair
▢ Poor
▢ Very Poor

Housing History

Researcher's Note-Source: Homeless Health Needs Audit sourced from Homeless Link and ACORN screening tool sourced from the Social Interventions Research and Evaluation Network (SIREN)

We would like to ask you some questions about your housing history. If you have had multiple stays in shelter, please report on the most recent one.

23. How many times have you stayed in shelter? ▢▢

24. When did you exit shelter (Month/Day/Year)? If you can't recall the exact date, please approximate around what time and round to the nearest month (e.g., 12/01/2024, 01/01/2025, 02/01/2025). ▢▢

25. During the last time you experienced homelessness with your child under age 5, how long were you living in shelter?

▢▢ weeks, months, years

26. How many family members lived with you while you were in shelter? ▢▢

27. Where are you and your family currently sleeping?
▢ Doubled-up or Sofa surfing – staying with friends/relatives
▢ Car
▢ Squatting
▢ In an emergency accommodation, e.g., HERRCS (Emergency Response and Relief Centers), night shelter, refuge
▢ Sleeping on the streets/park
▢ In a hostel, hotel, or supported accommodation, e.g., a faith-based organization
▢ Department of Homeless Services (DHS) shelter, e.g., Henry Street Settlement, WIN (Women in Need), Volunteers of America, Homes for the Homeless, HELP Family Services Corporation, Housing Works, or any provider on this list: https://www.nyc.gov/assets/dhs/downloads/pdf/dhs-provider-list-2024.pdf
▢ Domestic Violence (DV) shelter, e.g., Safe Horizon, Inc.

▢ Department of Housing Preservation and Development (HPD) shelter
▢ Owner-occupied home
▢ Rental Apartment/House/Room (no government subsidy)
▢ Rental Apartment/House/Room (with government subsidy)
▢ Other e.g., public housing, market rental, owner-occupied, private rented housing
-- please state

28. Thinking about the most recent time you became homeless, what was the main reason for this?

Give one primary reason and one secondary reason, if applicable. If you wish to select more than two reasons, you may select an additional reasonin **the third column**.	Primary Reason	Secondary Reason	Additional reason (Optional)
Parents/caregivers no longer able or willing to accommodate	▢	▢	▢
Other relatives or friends no longer able or willing to accommodate	▢	▢	▢
Non-violent relationship breakdown with partner	▢	▢	▢
Abuse or domestic violence	▢	▢	▢
Overcrowded housing	▢	▢	▢
Eviction or threat of eviction	▢	▢	▢
Rent or mortgage arrears	▢	▢	▢
Other debt related issues	▢	▢	▢

End of tenancy (social housing, private rented sector)	▢	▢	▢
Financial problems caused by benefits reduction	▢	▢	▢
Unemployment	▢	▢	▢
Anti-social behavior (ASB) or crime	▢	▢	▢
Drug or alcohol problems	▢	▢	▢
Mental health problems	▢	▢	▢
Physical health problems	▢	▢	▢
Leaving institutional care (e.g. hospital, prison, care, etc.)	▢	▢	▢
Administration burden	▢	▢	▢
Foreclosure on landlord's property	▢	▢	▢
Environment – House fire or flooding	▢	▢	▢
Other -- please state	▢	▢	▢

(Researcher's Note- For Question 28, the participant will be able to view the following descriptions below under each reason as requested by committees; please see below)

Descriptions

1. Parents/caregivers no longer able or willing to accommodate: **Ex. Lack of space in parents/caregivers' accommodations, financial or housing hardships faced by parents/caregivers, fractured relationship with parents/caregivers**

2. Other relatives or friends no longer able or willing to accommodate: **Ex. Lack of space in friends/relatives' accommodations, financial or housing hardships faced by relatives/friends, fractured relationship with friends/relatives**

3. Non-violent relationship breakdown with partner: **Ex. The ending of a relationship with a partner that resulted in losing housing shared with that person**

4. Abuse or domestic violence: **Ex. Fleeing housing shared with abusive partner/parent/caregiver/relative, etc.**

5. Overcrowded housing: **Ex. Lack of space in shared housing arrangement that became too unsafe/uncomfortable to continue living there**

6. Eviction or threat of eviction: **Ex. Being evicted or threatened with eviction from housing due to financial hardship, and/or other problems with landlord**

7. Rent or mortgage arrears: **Ex. Debt from coming up short and/or late with rent or mortgage payments (possibly multiple times)**

8. Other debt related issues: **Ex. Being unable to financially sustain oneself due to any (or a combination of) the following: medical debt, student loan debt, credit card debt, etc.**

9. End of tenancy (social housing, private rented sector): **Ex. Being unable to secure housing after the end of a lease**

10. Financial problems caused by benefits reduction: **Ex. Becoming disqualified from a housing voucher after a change in employment/income, losing access to government benefits such as EBT, SNAP, WIC, etc. after a change in eligibility**

11. Unemployment: **Ex. Being unable to financially sustain oneself due to lack of stable work, being unable to acquire a job in a timely manner after becoming terminated from one's job or a work contract concluding/workplace shutting down**

12. Anti-social behavior (ASB) or crime: **Ex. Facing violence and/or other crimes or the threat of them by individuals in your housing accommodation and/or community/neighborhood**

13. Drug or alcohol problems: **Ex. Facing substance abuse issues that resulted in financial/job loss through the inability to sufficiently access care for oneself**

14. Mental health problems: **Ex. Facing mental health problems that resulted in financial/job loss through the inability to sufficiently access care for oneself**

15. Physical health problems: **Ex. Facing physical health problems that resulted in financial/job loss through the inability to sufficiently access care for oneself**

16. Leaving institutional care (e.g. hospital, prison, care, etc.): **Ex. Inability to secure stable housing and other supportive resources that would've prevented homelessness after a long term stay in a hospital or post-incarceration**

17. Administration burden: **Ex. Experiencing too much difficulty/lack of support in accessing government resources such as benefits and vouchers that would prevent homelessness**

18. Foreclosure on landlord's property: **Ex. Being ordered to vacate your housing due to possession of property by a bank/lender as a result of landlord's financial problems**

19. Environment - House fire or flooding: **Ex. Housing being destroyed or severely damaged by a natural disaster**

Physical Environment

Researcher's Note- Source: NSCH, "Statutory homelessness in England: the experience of families and 16-17", and Kingfisher housing evaluation

We would like to ask you some questions about the environment you lived in during your time in shelter. If you have had multiple stays in shelter, please report on the most recent one.

29. How many bedrooms (including rooms used as bedrooms) were there in your shelter accommodation (not including kitchens & bathrooms)?

- ❑ One
- ❑ Two
- ❑ Three
- ❑ Four
- ❑ Five
- ❑ More than 5

30. Please list how many people were sharing a room used as a bedroom
Room 1: (insert number of people, child or adults)
Children (under 5 years old) ▢▢
Children (5-17 years old) ▢▢

Adults (18 years old or older) ▢▢

(Note: Option for participant to add another room)
Room 2: (insert number of people, children or adults)
Children (under 5 years old) ▢▢
Children (5-17 years old) ▢▢
Adults (18 years old or older) ▢▢

31. How often did you have trouble with dampness (e.g., condensation) or mold in your shelter accommodation?

- ❑ 1 = Never
- ❑ 2 = Rarely
- ❑ 3 = Sometimes
- ❑ 4 = Often
- ❑ 5 = Always

32. How often did you have trouble with vermin (e.g., mice, rats, fleas, bedbugs, or cockroaches) in your accommodation?

- ❑ 1 = Never
- ❑ 2 = Rarely
- ❑ 3 = Sometimes
- ❑ 4 = Often
- ❑ 5 = Always

33. How often did you have trouble with unsafe electrics? (For example, electrical units coming out of the wall and lack of baby/childproofing)

- ❑ 1 = Never
- ❑ 2 = Rarely
- ❑ 3 = Sometimes
- ❑ 4 = Often
- ❑ 5 = Always

34. Did you receive any sunlight in your shelter accommodation? (e.g., through windows or doors)
▢ No
▢ Yes

35. Was there space for your child to play active games (tag/playing with a ball, sit and ride toys or push toys) inside the shelter accommodation?
▢ No
▢ Yes

36. Did your shelter accommodation have a safe outdoor space (e.g., a garden or yard) where your child(ren) could exercise or play?
▢ No (Please also answer question 36a)
▢ Yes (Please also answer questions 36b and 36c)

36a.If no, did you have access to a park or a play area nearby?
▢ No
▢ Yes

36b. If yes, was your garden/yard shared or private?
▢ Shared
▢ Private

36c. If yes, in the last month of your stay in your accommodation, how often did your child(ren) play outside in your garden/yard?
- ❑ 1= Never
- ❑ 2= 1-3 times this month
- ❑ 3= Once a week
- ❑ 4= 2-3 times a week
- ❑ 5= 5-6 times a week
- ❑ 6= Every day

37. Did you share any of the following with people who were not members of your household? (Select all that apply)
▢ Kitchen
▢ Toilet and bathroom/shower facilities
▢ Clothes drying room

38. Did your shelter accommodation experience noise from neighbors or surroundings that interfered with your day-to-day life?	▢ No	▢ Yes
39. Could you access the internet from your shelter accommodation (e.g., broadband/data on your phone)?	▢ No	▢ Yes
40. If yes, was the internet connection sufficient to access essential services (e.g., virtual appointments, live news, important emails, etc.)?	▢ No	▢ Yes
41. Did you feel that your neighborhood was an unsafe place for your child to play in terms of criminal activity/anti-social behavior?	▢ No	▢ Yes
42. Your shelter accommodation was in such bad condition that you worried about your child(ren)'s safety.	▢ No	▢ Yes

43. In winter, you were able to keep your shelter accommodation warm enough.	☐ No	☐ Yes
44. In summer, you were able to keep your shelter accommodation cool enough.	☐ No	☐ Yes
45. You were able to properly ventilate the property (e.g., open the window, turn on a fan).	☐ No	☐ Yes
46. There was a supermarket within easy reach of your shelter accommodation.	☐ No	☐ Yes
47. There was overcrowding – too many people living in the property given its size.	☐ No	☐ Yes
48. There was nowhere suitable for child(ren) to do homework.	☐ No	☐ Yes
49. There was not enough space for smaller items such as toys, clothes, etc.	☐ No	☐ Yes
50. It caused arguments among the family group.	☐ No	☐ Yes
51. There was a lack of privacy.	☐ No	☐ Yes
52. There was a problem with passive smoking.	☐ No	☐ Yes

Social Environment

Researcher's Note- Source: HOME (Home Observation for Measurement of the Environment)

We are interested in your family's lifestyle and rules when you were living in the shelter system. Please think about the environment you lived in with your child(ren) under age 5 years old and respond to the following questions as best you can. Please select one answer per question or fill in the blanks when prompted. In the following questions, "child" refers only to your child(ren) who was under age 5 years old while you were both living in the shelter system.

53. How often did your **child** eat a meal with family members?
- ❑ 1=Never,
- ❑ 2=Once a month or less,
- ❑ 3=Once a week,
- ❑ 4=Several times a week,

- ❑ 5=Once a day,
- ❑ 6=More than once a day

54. How often did your family attend religious activities, including services?
    - ❑ Never
    - ❑ Once or twice a month
    - ❑ More than once a week
    - ❑ A few times a year
    - ❑ Once a week

55. How often did your whole family get together with relatives or friends?
    - ❑ 1=Never
    - ❑ 2=Once a year,
    - ❑ 3=A few times a year,
    - ❑ 4=Once a month,
    - ❑ 5=Two or three times a month,
    - ❑ 6=Once a week or more

56. About how often did your **child** have a chance to get out of the house?
    - ❑ 1=A few times a month or less,
    - ❑ 2=About once a week,
    - ❑ 3=A few times a week,
    - ❑ 4=Four or more times a week,
    - ❑ 5=Every day

57. How often did you or another adult in your home take your **child** to the grocery store?
    - ❑ 1=Hardly ever,
    - ❑ 2=Once a month,
    - ❑ 3=Once a week,
    - ❑ 4=Twice a week or more

58. How often did your **child** spend time with you or another adult in your home doing outdoor activities?
    - ❑ 1=Never,
    - ❑ 2=A few times a year or less,
    - ❑ 3=Once a month,
    - ❑ 4=Once a week,
    - ❑ 5=At least four times a week,
    - ❑ 6=Once a day or more often

59. How often did you or another adult in your home get a chance to read stories to your **child**?
    - ❑ 1=Never,
    - ❑ 2=Several times a year,
    - ❑ 3=Several times a month,

- ❑ 4=Once a week,
- ❑ 5=About 3 times a week,
- ❑ 6=Every day

60. About how many children's books did your child have?
- ❑ 1=None,
- ❑ 2=1-2 books
- ❑ 3=3-9 books
- ❑ 4=10-19 books
- ❑ 5=20 or more books

61a. How many, if any, push or pull toys did your child have (may be shared with sister or brother)?
Please write in number of push/pull toys. NUMBER OF TOYS = _________(Open-ended response)

61b. How many cuddly, soft, or role-playing toys (like a doll) did your child have (may be shared with sister or brother)?
Please write in number of soft toys. NUMBER OF TOYS = ___________(Open-ended response)

61c. About how many hours were the TV or other screens in your home in used each day?
Please write in hours per day. HOUR PER DAY = ____________(Open-ended response)

62. Children seem to demand attention when their parents are busy, doing housework, for example. How often did you talk to your child while you were working?
- ❑ 1=Never,
- ❑ 2=Rarely,
- ❑ 3=Sometimes,
- ❑ 4=Often,
- ❑ 5=Always

Child Health

Researcher's Note- Source: 2019 NYC KIDS Survey, 2024 National Survey of Children's Health (including 2023 NSCH- ACEs section), 2024 U.S. Household Food Security Survey Module

We are interested in your child's physical, social, and mental health. Please reflect on these aspects of your child's health and respond to the following questions as best you can. Please select one answer per question UNLESS otherwise specified, or fill in the blanks. <u>In the ALL following questions, "child" refers only to your child(ren) who was under age five years old at the time you lived in the shelter system.</u> Please report only on your child(ren) who was under age five years old while you lived in the shelter system.

63. Has anyone in **your family** (e.g., your parents, grandparents, aunts, uncles, cousins, including yourself) been diagnosed or have a history with any of the following? (Select ALL that apply)

- ❑ Allergies (including food, drug, insect, seasonal or other)
- ❑ Arthritis
- ❑ Asthma
- ❑ Autism Spectrum Disorder
- ❑ Blood Disorders (such as Sickle Cell Disease, Thalassemia, or Hemophilia)
- ❑ Cancer
- ❑ Cerebral Palsy Clotting Disorder
- ❑ Cystic Fibrosis
- ❑ Dementia/Alzheimer's
- ❑ Diabetes
- ❑ Epilepsy or Seizure Disorder
- ❑ Frequent or Severe Headaches, including Migraine
- ❑ Gastrointestinal Disorder
- ❑ Heart Disease
- ❑ High Cholesterol
- ❑ Hypertension or High Blood Pressure
- ❑ Intellectual Disability or Learning Disorder
- ❑ Kidney Disease
- ❑ Lung Disease
- ❑ Osteoporosis
- ❑ Psychological Disorder
- ❑ Septicemia
- ❑ Stroke/Brain Attack
- ❑ Sudden Death
- ❑ Tuberculosis (TB)
- ❑ Unknown Disease
- ❑ Other (please specify)

Researcher's Note: (Participant can put in another entry if more than one child is under age 5.)

64. In general, how would you describe **your child's** health WHILE LIVING IN shelter?

- ❑ 1=Excellent
- ❑ 2=Very Good
- ❑ 3=Good
- ❑ 4=Fair
- ❑ 5=Poor

65. In general, how would you describe **your child's** health AFTER leaving shelter?

- ❑ 1=Excellent

- ☐ 2=Very Good
- ☐ 3=Good
- ☐ 4=Fair
- ☐ 5=Poor

66. Has your child had FREQUENT or CHRONIC difficulty with any of the following WHILE LIVING IN SHELTER as well as AFTER LEAVING SHELTER? (Select ALL that apply)

**List of Conditions**	**Select condition(s) that apply WHILE LIVING IN shelter**	**Select condition(s) that apply AFTER leaving shelter**
Breathing or other respiratory problems (such as wheezing or shortness of breath)	☐	☐
Eating or swallowing because of a health condition	☐	☐
Digesting food, including stomach/intestinal problems, constipation, or diarrhea	☐	☐
Repeated or chronic physical pain, including headaches or other back or body pain	☐	☐
Using his or her hands	☐	☐
Coordination or moving around	☐	☐
Toothaches or Bleeding gums	☐	☐

67. Has a doctor or other health care provider EVER told you that **your child** has any of the following (Select ALL that apply) **List of Conditions**	**Select Condition(s) that apply**
Allergies (including food, drug, insect, or other)	☐
Arthritis	☐
Asthma	☐
Blood Disorders (such as Sickle Cell Disease, Thalassemia, or Hemophilia)	☐

Brain Injury, Concussion or Head Injury	☐
Cerebral Palsy	☐
Cystic Fibrosis	☐
Diabetes	☐
Epilepsy or Seizure Disorder	☐
Heart Condition	☐
Frequent or Severe Headaches, including Migraine	☐
Tourette Syndrome	☐
Oppositional defiant disorder, conduct disorder, or any other behavioral or conduct problem	☐
Developmental Delay (For example, Delays in rolling over, sitting up, crawling and walking. Trouble with fine motor skills. Problems understanding what others say. Trouble with problem-solving. Issues with social skills. Problems talking or talking late. Difficulty remembering things)	☐
Intellectual Disability (For example, Down's Syndrome, Fragile X Syndrome, Fetal Alcohol Syndrome, and Prader-Willi Syndrome)<br>OR<br>Learning disorder (For example, Dyslexia, Dyscalculia, and Dysgraphia)	☐
Speech or Other Language Disorder (For example, Aphasia, Articulation Disorder, Phonological Disorder, Stuttering, Speech Sound Disorders, Development Language Disorder, and Dysarthria)	☐
Depression, Anxiety, or Adjustment Disorder	☐
Attention deficit disorder or attention deficit or hyperactivity disorder, that is, A.D.D. or A.D.H.D.	☐

	If SELECTED, then answer these additional questions:<br><br>Did your child receive medication?<br><br>▢<br><br>Did your child receive behavioral treatment, such as training or an intervention that you or your child received to help with his or her behavior WHILE LIVING IN shelter?<br><br>▢
Autism, Asperger's disorder, pervasive developmental disorder (PDD), or autism spectrum disorder (ASD)	▢<br><br>If SELECTED, then answer these additional questions:<br><br>Did your child receive medication?<br><br>▢<br><br>Did your child receive behavioral treatment, such as training or an intervention that you or your child received to help with his or her behavior WHILE LIVING IN shelter?<br><br>▢
Overweight or Obese	▢
Other condition(s) not listed (please specify)<br><br>_________________________	▢

68. How often did your child's health conditions or problems (selected above) affected his or her ability to do things other children his or her age do WHILE LIVING IN shelter?

- ❑ 1 = Your child does not have any conditions
- ❑ 2 = Never
- ❑ 3 = Sometimes
- ❑ 4 = Usually
- ❑ 5= Always

69. To what extent do your child's health conditions or problems affect his or her ability to do things?

- ❑ 1 = Your child does not have any conditions
- ❑ 2 = Very little

- ❑ 3 = Somewhat
- ❑ 4 = A great deal

70. SINCE YOUR CHILD WAS BORN, how often has it been very hard to cover the basics, like food or housing, on your family's income?

- ❑ 1 = Never
- ❑ 2 = Rarely
- ❑ 3 = Somewhat often
- ❑ 4 = Very often

71. The following questions are about events that may have happened during your child's life. These things can happen in any family, but some people may feel uncomfortable with these questions. You may skip any questions you do not want to answer (Please select one response per line).

Events that may have happened during your child's life	YES	NO
To the best of your knowledge, has your child EVER experienced any of the following? Parent or guardian divorced or separated	▢	▢
To the best of your knowledge, has your child EVER experienced any of the following? Parent or guardian death.	▢	▢
To the best of your knowledge, has your child EVER experienced any of the following? Parent or guardian served time in jail	▢	▢
To the best of your knowledge, has your child EVER experienced any of the following? Saw or heard parents or adults slap, hit, kick, punch one another in the home	▢	▢
To the best of your knowledge, has your child EVER experienced any of the	▢	▢

following? Was a victim of violence or witnessed violence in his or her neighborhood		
To the best of your knowledge, has your child EVER experienced any of the following? Lived with anyone who was mentally ill, suicidal, or severely depressed	☐	☐
To the best of your knowledge, has your child EVER experienced any of the following? Lived with anyone who had a problem with alcohol or drugs	☐	☐
To the best of your knowledge, has your child EVER experienced any of the following? Treated or judged unfairly because of his or her race or ethnic group	☐	☐
To the best of your knowledge, has your child EVER experienced any of the following? Treated or judged unfairly because of a health condition or disability	☐	☐
To the best of your knowledge, has your child EVER experienced any of the following? Treated or judged unfairly because of their sexual orientation or gender identity	☐	☐

72. For these statements, please tell us whether the statement was often true, sometimes true, or never true while you were living in the shelter system.

72a. The first statement is, “The food that (I/we) bought just didn’t last, and (I/we) didn’t have money to get more.” Was that often, sometimes, or never true for (you/your former shelter household) while living in the shelter system?

- ❑ Often true
- ❑ Sometimes true
- ❑ Never true

72b. “(I/we) couldn’t afford to eat balanced meals.” Was that often, sometimes, or never true for (you/your family) while living in the shelter system?

- ❑ Often true
- ❑ Sometimes true
- ❑ Never true

72c. While living in the shelter system, did (you/you or other adults in your household) ever cut the size of your meals or skip meals because there wasn't enough money for food?

- ❑ NO
- ❑ YES

72d. How often did this happen—almost every month, some months but not every month, or in only 1 or 2 MONTHS?

- ❑ Almost every month
- ❑ Some months but not every month
- ❑ Only 1 or 2 months

72e. While living in the shelter system, did you ever eat less than you felt you should because there wasn't enough money for food?

- ❑ YES
- ❑ NO

72f. While living in the shelter system, were you ever hungry but didn't eat because there wasn't enough money for food?

- ❑ YES
- ❑ NO

73. Does your child have any of the following?

73a. Deafness or problems with hearing
- ❑ YES
- ❑ NO

73b. Blindness or problems with seeing, even when wearing glasses
- ❑ YES
- ❑ NO

74. How would you describe the condition of your child’s teeth?

- ❑ 1 = Excellent
- ❑ 2 = Very good

- ❑ 3 = Good
- ❑ 4 =Fair
- ❑ 5 = Poor
- ❑ 6 = Your child does not have any teeth

75. Fluoride is applied as a treatment on teeth to prevent cavities. Has your child ever had fluoride treatment? This may have happened at your dental office, pediatrician's office, or through a school dental program.

- ❑ YES
- ❑ NO

76. How well do each of the following phrases describe your child **while living in shelter** (Please select one answer per phrase)?

List of Phrases	DEFINITELY TRUE	SOMEWHAT TRUE	NOT TRUE
Your child is affectionate and tender with you	☐	☐	☐
Your child bounces back quickly when things do not go his or her way	☐	☐	☐
Your child shows interest and curiosity in learning new things	☐	☐	☐
Your child smiles and laughs a lot	☐	☐	☐

77. Did your child receive any treatment or counseling from a mental health professional WHILE LIVING IN SHELTER? Mental health professionals include psychiatrists, psychologists, psychiatric nurses, and clinical social workers.

- ❑ Yes
- ❑ No, but your child needed to see a mental health professional
- ❑ No, your child did not need to see a mental health professional

78. Did your child see a specialist other than a mental health professional WHILE LIVING IN SHELTER?

- ❑ Yes
- ❑ No, but your child needed to see a specialist
- ❑ No, your child did not need to see a specialist

For the following questions, I would like you to think back to when your child was a newborn.

79. Was your child born more than 3 weeks before his or her due date?

- ❑ YES
- ❑ NO

80. How much did he or she weigh when born? ▢▢ (if you can't remember, please do your best to estimate)

81. When your child was a newborn, was he or she ever breastfed or fed breast milk?

- ❑ YES
- ❑ NO

82. How old was your child when the child completely stopped breastfeeding or being fed breastmilk? (Select your preferred choice of time measurement and fill in the blank)

- ❑ My child stopped breastfeeding... (Select your preferred choice of time measurement and fill in the blank)
  - o NUMBER OF YEARS
  - o OR
  - o NUMBER OF MONTHS
  - o OR
  - o NUMBER OF WEEKS
  - o OR
  - o NUMBER OF DAYS
- ❑ STILL BREASTFEEDING

83. A home visitor is a nurse, a health care worker, a social worker, or other person who works for a program that helps mothers of newborns in the mother's home. Thinking back to the first month after your child was born, did a home visitor come to your home to help the family learn how to take care of the family and your new baby?

- ❑ YES
- ❑ NO
- ❑ WAS NOT CHILD'S PARENT DURING FIRST MONTH OF LIFE

84. Did your child's doctors or other health care providers ask if you have concerns about your child's learning, development, or behavior WHILE LIVING IN SHELTER?

- ❑ YES
- ❑ NO

85. Has your child EVER received special services to meet his or her developmental needs such as speech, occupational, or behavioral therapy?

- ❑ NO
- ❑ YES

- ☐ how old was your child when he or she began receiving these special services?
  - ☐ NUMBER OF YEARS _____
  - ☐ NUMBER OF MONTHS _____
- ☐ Is your child CURRENTLY receiving special services
  - ☐ YES
  - ☐ NO

86. Was there any time when your child needed health care but it was not received WHILE LIVING IN SHELTER?

- ☐ NO
- ☐ YES
  - What types of care were not received (Select ALL that apply)
    - ☐ Medical Care
    - ☐ Dental Care
    - ☐ Vision Care
    - ☐ Hearing Care
    - ☐ Mental Health Services
    - ☐ Other, specify: _____
  - Which of the following contributed to your child not receiving needed health services (Select ALL that apply):
    - ☐ Your child was not eligible for the services
    - ☐ The services your child needed were not available in your area
    - ☐ There were problems getting an appointment when your child needed one
    - ☐ There were problems with getting transportation or child care
    - ☐ The (clinic/doctor's) office wasn't open when your child needed care
    - ☐ There were issues related to cost
    - ☐ Stigmatism (Negative association/relationship with health services)
    - ☐ Prefer Not to Say

87. How often were you frustrated in your efforts to get services for your child WHILE LIVING IN SHELTER?

- ☐ 1 = Never
- ☐ 2 = Sometimes
- ☐ 3 = Usually
- ☐ 4 = Always

Parental Mental Health

Researcher's Note -Source: Patient Health Questionnaire eight-item depression measure (PHQ-8), General Anxiety Disorder (GAD-7), and Adverse Childhood Experiences International Questionnaire (ACE-IQ) D1. 4 Section D: Guidance for Analyzing ACE-IQ (Binary Version, WHO)

We are interested in your mental health. Please think about your mental health and its origins as delicately as you can. Respond to the following questions as best you can. Please select one answer per question. These questions are strictly confidential and will NOT be shared with your providers or services.

88. When living in shelter, how often were you bothered by any of the following problems? Please select one response per line.

List of possible problems you have been bothered by	NOT AT ALL	SEVERAL DAYS	MORE THAN HALF THE DAYS	NEARLY EVERY DAY
a. Little interest or pleasure in doing things	☐	☐	☐	☐
b. Feeling down, depressed, or hopeless	☐	☐	☐	☐
c. Trouble falling or staying asleep, or sleeping too much	☐	☐	☐	☐
d. Feeling tired or having little energy	☐	☐	☐	☐
e. Poor appetite or overeating	☐	☐	☐	☐
f. Feeling bad about yourself-or that you are a failure or have let yourself or your family down	☐	☐	☐	☐
g. Trouble concentrating on things, such as reading the newspaper or watching television.	☐	☐	☐	☐
h. Moving or speaking so slowly that other people could have noticed? Or the opposite - being so fidgety or restless that you have been	☐	☐	☐	☐

moving around a lot more than usual.				

89. When living in shelter, how often were you bothered by the following problems? Please select one response per line.

List of possible problems you have been bothered by	NOT AT ALL	SEVERAL DAYS	MORE THAN HALF THE DAYS	NEARLY EVERY DAY
a. Feeling nervous, anxious, or on edge	▢	▢	▢	▢
b. Not being able to stop or control worrying	▢	▢	▢	▢
c. Worrying too much about different things	▢	▢	▢	▢
d. Trouble relaxing	▢	▢	▢	▢
e. Being so restless that it is hard to sit still	▢	▢	▢	▢
f. Becoming easily annoyed or irritable	▢	▢	▢	▢
g. Feeling afraid, as if something awful might happen	▢	▢	▢	▢

The following questions are about events that may have happened during **your childhood**. These things can happen in any family, but some people may feel uncomfortable with these questions.

90. Please select **YES or NO** if **you** experienced one or more events in the following categories; some categories contain more than one event, but you do not need to specify what event you experienced. You may skip any questions you do not want to answer.

Category: Physical abuse<br>• Did a parent, guardian or other household member spank, slap, kick, punch or beat you up?<br><br>• Did a parent, guardian or other household member hit or cut you with an object, such as a stick (or cane), bottle, club, knife, whip etc?	❑ YES<br>❑ NO
Category: Emotional abuse<br>• Did a parent, guardian or other household member yell, scream or swear at you, insult or humiliate you?<br>• Did a parent, guardian or other household member threaten to, or actually, abandon you or throw you out of the house?	❑ YES<br>❑ NO
Category: Contact sexual abuse<br>• Did someone touch or fondle you in a sexual way when you did not want them to?<br>• Did someone make you touch their body in a sexual way when you did not want them to?<br>• Did someone attempt oral, anal, or vaginal intercourse with you when you did not want them to?<br>• Did someone actually have oral, anal, or vaginal intercourse with you when you did not want them to?	❑ YES<br>❑ NO
Category: Alcohol and/or drug abuser in the household<br>• Did you live with a household member who was a problem drinker or alcoholic, or misused street or prescription drugs?	❑ YES<br>❑ NO
Category: Incarcerated household member<br>• Did you live with a household member who was ever sent to jail or prison?	❑ YES<br>❑ NO
Category: Household member treated violently<br>• Did you see or hear a parent or household member in your home being yelled at, screamed at, sworn at, insulted or humiliated?	❑ YES<br>❑ NO

• Did you see or hear a parent or household member in your home being slapped, kicked, punched or beaten up?<br>• Did you see or hear a parent or household member in your home being hit or cut with an object, such as a stick (or cane), bottle, club, knife, whip etc.?	
<u>Category: One or no parents, parental separation or divorce</u><br>• Were your parents ever separated or divorced?<br>• Did your mother, father or guardian die?	❑ YES<br>❑ NO
<u>Category: Emotional neglect</u><br>• Did your parents/guardians understand your problems and worries?<br>• Did your parents/guardians really know what you were doing with your free time when you were not at school or work?	❑ YES<br>❑ NO
<u>Category: Physical neglect</u><br>• Did your parents/guardians not give you enough food even when they could easily have done so?<br>• Were your parents/guardians too drunk or intoxicated by drugs to take care of you?<br>• Did your parents/guardians not send you to school even when it was available?	❑ YES<br>❑ NO
<u>Category: Bullying</u><br>• Were you bullied?	❑ YES<br>❑ NO
<u>Category: Community violence</u><br>• Did you see or hear someone being beaten up in real life?<br>• Did you see or hear someone being stabbed or shot in real life?<br>• Did you see or hear someone being threatened with a knife or gun in real life?	❑ YES<br>❑ NO
<u>Category: Collective Violence</u>	❑ YES

• Were you forced to go and live in another place due to any of these events?<br>• Did you experience the deliberate destruction of your home due to any of these events?<br>• Were you beaten up by soldiers, police, militia, or gangs?<br>• Was a family member or friend killed or beaten up by soldiers, police, militia, or gangs?	❑ NO

Thank you for taking the time to complete our survey.

Please leave your email address if you would like us to send you a gift card and follow up.

Please indicate **YES or NO** if you would like to be contacted to participate in an interview after the survey. Interviews last approximately 30-45 minutes, and we will give you a $40 gift card for your time.

- ❑ YES
- ❑ NO
